# MiR-125a-5p regulates the radiosensitivity of laryngeal squamous cell carcinoma via HK2 targeting through the DDR pathway

**DOI:** 10.3389/fonc.2024.1438722

**Published:** 2024-08-19

**Authors:** Qiwei Wang, Lijun Tan, Yuanhang Lv, Tianjiao Yu, Yuan Chang, Jiangtao Liu, Yanan Sun

**Affiliations:** ^1^ Department of Otolaryngology, Head and Neck Surgery, Harbin Medical University, Harbin, Heilongjiang, China; ^2^ Department of Oncology, The First Affiliated Hospital of Harbin Medical University, Harbin, Heilongjiang, China; ^3^ Department of Otolaryngology, Head and Neck Surgery, Zhengzhou Central Hospital Affiliated Zhengzhou University, Zhengzhou, Henan, China; ^4^ Department of Otolaryngology, Head and Neck Surgery, The First Affiliated Hospital of Harbin Medical University, Harbin, Heilongjiang, China; ^5^ Department of Otolaryngology, Head and Neck Surgery, The Second Affiliated Hospital of Harbin Medical University, Harbin, Heilongjiang, China

**Keywords:** radiosensitivity, head and neck squamous cell carcinoma, miR-125a-5p, laryngeal squamous cell carcinoma, HK2

## Abstract

**Objective:**

To determine the function of miR-125a-5p in laryngeal squamous cell carcinoma (LSCC), its correlation with radiation sensitivity, and the underlying regulatory mechanism.

**Materials and methods:**

We conducted the analysis on the correlation between miR-125a-5p and head and neck squamous cell carcinoma (HNSCC) using data obtained from The Cancer Genome Atlas (TCGA). The putative gene targeted by miR-125a-5p has been identified as HK2, while the expression levels of miR-125a-5p and HK2 were measured in laryngeal cancer tissues and cells using RT-PCR. MiR-125a-5p and HK2 were introduced into the lentiviral vector and the vector was used to transfect AMC-HN-8 cells. The roles of miR-125a-5p and HK2 in LSCC and on radiosensitivity were determined by evaluating cell growth, examining colony formation, analyzing flow cytometry, and utilizing the single hit multi-target model. Western blotting was used to measure H2AX and rH2AX levels in the DNA damage response (DDR) pathway. The validation of the interaction between miR-125a-5p and HK2 was conducted through the dual-luciferase assay. To further confirm the association between miR-125a-5p and HK2, as well as its influence on radiosensitivity, rescue experiments were performed.

**Results:**

The expression of miR-125a-5p is downregulated in LSCC, while upregulating its expression could suppress cell growth, induce apoptosis, and enhance radiosensitivity. Additionally, HK2 exhibited high expression in LSCC and the biological function was opposite to miR-125a-5p. Western blotting analysis revealed that miR-125a-5p increased rH2AX levels and decreased H2AX levels, conversely, HK2 had the opposite effect on miR-125a-5p. These findings suggested that HK2 may serve as the target gene of miR-125a-5p. The double luciferase assay confirmed the binding of HK2 to miR-125a-5p, and rescue trials confirmed the role of miR-125a-5p in regulating the effects and radiation sensitivity of LSCC by targeting HK2 via the DDR pathway.

**Conclusion:**

By targeting HK2 and impacting the DDR pathway, miR-125a-5p has been found to inhibit cellular proliferation, enhance apoptosis, and heighten radiosensitivity in LSCC.

## 1 Introduction

Globally, laryngeal squamous cell cancer (LSCC) is a common type of cancer that is strongly linked to tobacco and alcohol consumption (1). Symptoms of LSCC include hoarseness, pain, difficulty swallowing, and breathing problems. Despite clinical therapy advancements and the use of multiple treatments, the mortality of patients with LSCC remains high. The treatment options for LSCC include chemotherapy, surgery, and radiation therapy. Radiotherapy is an important non-surgical treatment for this type of cancer and improving its effectiveness can greatly benefit patients.

The influence of microRNAs (miRNAs) on the modulation of tumor radiation response is significant. These small RNA molecules effectively regulate gene expression in LSCC through processes such as mRNA degradation and transcriptional inhibition (2). MiRNAs affect tumor growth, invasion, apoptosis, sensitivity to chemotherapy, and response to radiation therapy. Cancer-associated miRNAs exert the significant influence on the progression and metastasis of diverse cancer types, including LSCC. MiR-196b promotes LSCC through PCDH-17 targeting, miR-370 through FoxM1 targeting, and the interaction between miR-1246 and THBS1 promotes LSCC (3–5).

Scholars have extensively researched the role of the miR-125 family in tumorigenesis. This family of microRNAs is implicated in regulating tumor proliferation, apoptosis, metastasis, energy metabolism, angiogenesis, and modulating the sensitivity of tumors to radiotherapy and chemotherapy (6, 7). Upregulation of miR-125a-5p promotes the proliferation, migration, and invasion of head and neck squamous cell carcinoma cell lines by promoting C-C chemokine receptor 7 expression (8). Conversely, elevated miR-125b-5p is correlated with a favorable prognosis in LSCC and suppresses malignancy and metabolic disorder by targeting MAP3K9 (9). However, there is limited understanding regarding the specific contributions made by individual members of the miR-125 family to LSCC. Therefore, the aim of our study is to elucidate the precise roles played by different members of this family in LSCC.

## 2 Materials and methods

### 2.1 Data collection from The Cancer Genome Atlas

Prior to this study, we conducted a thorough literature review and found that the miR-125 family is closely associated with tumorigenesis (6, 7). Hence, we obtained and analyzed miRNA-seq data (in the RPM format) sourced from TCGA-HNSC(head and neck squamous cell carcinoma) project from the TCGA database (https://portal.gdc.cancer.gov). We identified matched normal and tumor sample pairs based on numerical identifiers. In addition, clinical information for miR-125a-5p, including overall survival (OS) event, clinical stage, histologic grade, pathologic stage, lymph node neck dissection, lymphovascular invasion, and radiation therapy was extracted from the TCGA database.

### 2.2 Identification of genetic targets

StarBase (http://starbase.sysu.edu.cn/), miRDB (http://www.mirdb.org/), and TarBase v.8 (https://dianalab.e-ce.uth.gr/html/diana/web/index.php?r=tarbasev8) were used to predict target genes. The construction of the protein-protein interaction (PPI) network involved merging datasets from the STRING database (version 11.5)(https://string-db.org/cgi/input.pl). The network was visualized using Cytoscape software version 3.8.0, with support from the CytoHubba plugin (10), to identify key genes. Potential sites for binding were predicted by utilizing the TargetScan database( https://www.targetscan.org).

### 2.3 Cell lines and human samples

Between January 2020 and January 2021, 44 tissue samples were collected from the Department of Ear, Nose, and Throat Surgery at the Second Affiliated Hospital of Harbin Medical University, comprising both malignant and adjacent non-malignant tissues. Ethical approval was obtained from the Medical Ethics Committee of the Second Affiliated Hospital of Harbin Medical University (KY2017-047). The cell lines including Tu212, AMC-HN-8, hep-2, and BEAS-2B, were obtained from Fenghui Biology.

### 2.4 Cell transfection

AMC-HN-8 cells were digested the day prior to transfection and were in a robust logarithmic growth stage. The GSK lentivirus vector was prepared according to the manufacturer’s instructions (Sangon Biotech Engineering Co., Ltd, Shanghai, China). The cells were maintained in a standard culture medium for 72 h. Following incubation, transfection efficiency was assessed using fluorescence microscopy. Cells were harvested from samples with ≥80% transfection efficiency, and stably transfected cells were selected using puromycin (2 µg/mL). The test groups consisted of the parental (untransfected), Ad-control, Si-control (blank vector group), Ad-miR-125a-5p (miR-125a-5p overexpression), Ad-HK2 (HK2 overexpression), Si-HK2 (HK2 silencing), and Ad-miR-125a-5p+HK2 (co-transfection of HK2 and miR-125a-5p) groups.

### 2.5 Immunohistochemistry

Immunohistochemical analysis was performed to evaluate HK2 expression in LSCC tissues. During surgery, 44 sets of laryngeal cancer and surrounding healthy tissues were obtained, sliced into 4 mm circles, and stored in 4% PFA overnight. Subsequently, tissues were dehydrated, embedded into blocks, sectioned, and underwent antigen retrieval. Next, the tissue sections were blocked using, and incubated with antibodies. DAB chromogenic solution was administered, and microscopic examinations were conducted using PBS. Image analysis was performed using ImageJ software to determine the mean optical density (MOD), where MOD= IntDen/Area. The IntDen signifies the cumulative optical density within a specified range, whereas the Area represents the total area encompassed by the image.

### 2.6 RT-PCR

RNA was isolated from AMC-HN-8 cells and LSCC tissues using TRIzol reagent (Invitrogen, Carlsbad, CA, USA), and reversed-transcribed into cDNA using a cDNA synthesis kit (74134 and 74136, Qiagen, Germany). The primer sequences used were U6 forward: 5’-CTCGCTTCGGCAGCACA-3’, U6 reverse: 5’-AACGCTTCACGAATTTGCGT-3’, GAPDH forward: 5’-CGGATTTGGTCGTATTGGG-3’, GAPDH reverse: 5’-CCTGGAAGATGGTGATGGG-3’, miR-125a-5p forward: 5’-CGCCGTCCCTGAGACCCTTTAAC-3’, miR-125a-5p reverse: 5’-ATCCAGTGCAGGGTCCGAGG-3’, HK2 forward: 5’-AAGGCTTCAAGGCATCTG-3’, and HK2 reverse: 5’-CCACAGGTCATCATAGTTCC-3’. RT-PCR analysis was performed using the MonAmpTM ChemoHS qPCR Mix detection kit, according to the manufacturer’s instructions, with GAPDH or U6 as internal reference genes. Analysis of the RT-qPCR data was conducted using the 2^−ΔΔCt^ technique.

### 2.7 CCK-8 assay

Following 24, 48, 72, and 96 h incubation, each cell-containing well was treated with 10 µL CCK-8 reaction solution for 2 h. Subsequently, sample absorbance was measured at 450 nm using an ELISA kit (Shanghai boke BIotechnology Co., Ltd. Shanghai, China) and a microplate reader.

### 2.8 Colony formation assay

AMC-HN-8 cells were cultured at densities of 500, 1 000, 2 000, 4 000, 8 000, and 16 000 cells per well, followed by exposure to X-ray radiation at doses of 0, 2, 4, 6, 8, and 10 Gy. The viable cells were methanol-fixed for 15 min and subsequently stained with Giemsa reagent for 20 min. Finally, the cells were visualized, images were captured, and the number of cloned cells was counted. The surviving fraction (SF) of the experimental groups (exposure to various radiation doses) was relative to that of the control group (0 Gy). Plating efficiency (PE) = (number of colonies of untreated cells/number of seeded cells)×100%, SF = number of clones in the experimental group/(initial number of seeded cells×PE). The k and N values were analyzed using GraphPad Prism software (version 4.0), with the single hit multi-target model: SF=1-(1-e^-kD^)^N^. Then we determined D_0_, D_37_, and the sensitivity enhancement ratio (SER). The average lethal dose, represented by D_0_, D_0_ = 1/k, is indicative of higher radioresistance with larger D_0_ values. Using the single hit multi-target model, we calculated the quasi-threshold dose (D_q_=ln(N×D_0_)) and D_37_ (the radiation dose that results in a cell survival fraction of 37%, D_37_=D_0_+D_q_). SER refers to changes in both the shape and parameters of the cell survival curve after treatment with different factors before irradiation, reflecting alterations in cellular radiosensitivity. The magnitude of SER indicated increased sensitivity towards radiation following treatment with the reagents: SER=D_q_(the control group without treatment)/D_q_ (the treated experimental group).

### 2.9 Western blotting

Protein extraction from both tissues and cells was performed using RIPA buffer (Solarbio PMSF, Beijing, China). The protein samples were combined with gel buffer containing sodium dodecyl sulfate-polyacrylamide and subsequently transferred onto nitrocellulose membranes following the process of electrophoresis. After washing with TBS-T, the membrane was blocked with 10% bovine serum albumin solution in TBS-T buffer. The membrane was incubated at room temperature for 2 h and then incubated overnight with specific primary antibodies against HK2 (ProSci, 1:1000), rH2AX (Microporous, 1:1000), H2AX (Abcam, 1:1000), and GAPDH (ZsBio, 1:5000). The membrane was then washed three times with TBS-T solution and treated with a secondary antibody (Zsbio, 1:5000). Following additional TBS-T washes, proteins were detected using a highly sensitive ECL chemiluminescence kit and visualized using a gel imaging system. Protein band intensity was quantitatively assessed using ImageJ software, with GAPDH as the internal reference.

### 2.10 Flow cytometry

The number of cells in the suspension was adjusted to 1 million cells/mL using a binding buffer. The cell mixture (100 µL) was combined with 5 µL of Annexin V and 10 µL of PI, followed by incubation 37°C, in the dark, for 20 min. Apoptosis was assessed using CytoFLEX flow cytometry, and cellular data was collected for apoptosis rate analysis using the CytExpert software system.

### 2.11 Dual luciferase assay

Transfection was performed using the Lipofectamine 2000 transfection reagent (Invitrogen), according to the manufacturer’s instructions. AMC-HN-8 cells were co-transfected with either wild-type or mutant reporter vectors (HK2-wt or HK2-mut) and either an miR-125a-5p mimic or miR-control. Following transfection, 100 µL of cellular lysate was immobilized on the lockwell maxisorp detection plate, and firefly luminescence was quantified using ELISA. Subsequently, 50 µL of Dual-Glo^®^ Stop & Glo^®^ Reagent (Promega, Beijing, China) was added into each well, and renilla luminescence was measured over 2 h. Luciferase expression was measured by dividing the firefly luciferase value by the renilla luciferase value in the same sample well.

### 2.12 Statistical analysis

R software (version 4.2.1) was used for data analysis, and appropriate statistical methods were selected for analysis considering the characteristics of the data format, utilizing stats (version 4.2.1) and car (version 3.1-0) packages, and visualizing the data using ggplot2 (version 3.3.6). Various statistical tests were used to analyze the data collected from the TCGA database, including the paired sample t-test to examine the expression discrepancies among the members of the miR-125 family in HNSCC, Wilcoxon rank sum test for OS event and lymphovascular invasion, t-test for radiation therapy, one-way ANOVA for clinical stage and pathological stage analysis, the Kruskal–Wallis test for histologic grade examination and lymph node neck dissection, and ROC analysis for radiation therapy diagnosis assessment. Immunohistochemical and RT-PCR tissue findings were analyzed using the Wilcoxon rank-sum test, whereas cell PCR outcomes and luciferase reporter gene experiments were assessed using t-tests. CCK-8 results were subjected to two-factor repeated measurement ANOVA. Colony formation and flow cytometry measurements were evaluated using one-way ANOVA, and protein expression was measured via western blotting, analysis was performed using a paired sample t-test. Data visualization was performed using ggplot2, with the significance level set at *P*<0.05.

## 3 Results

### 3.1 Identification of HNSCC-associated genes

**Figure 1:**
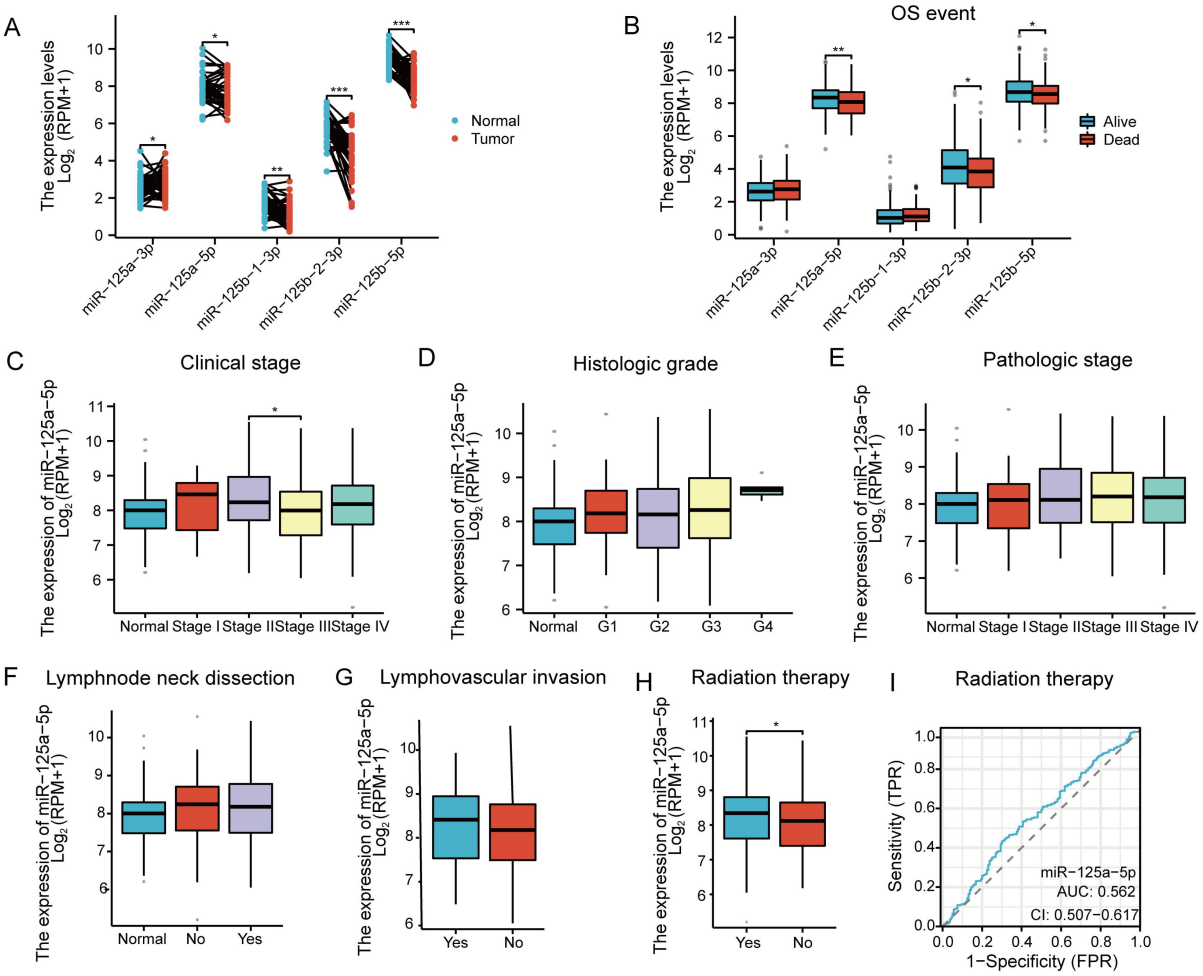
Gene screening and clinical relevance analysis in HNSCC: **(A)** Expression analysis of the miR-125 family members in HNSCC based on the data obtained from the TCGA database. **(B)** Analysis of Overall Survival(OS) events associated with the miR-125 family members. The analysis considered factors such as **(C)** clinical stage, **(D)** histologic grade, **(E)** pathologic stage, **(F)** lymphnode neck dissection, **(G)** lymphovascular invasion and **(H)** Radiation therapy. **(I)** ROC curve representing the diagnostic potential of miR-125a-5p in radiation therapy. (^*^ *P*<0.05, ^**^ *P*<0.01, and ^**^ *P*<0.001).

Paired sample analysis of HNSCC data from the TCGA database revealed that the miR-125 family is significantly differentially expressed in HNSCC (**Figure 1A**). Specifically, miR-125a-5p was downregulated in HNSCC, with the tumor group exhibiting significantly lower expression than the normal group (**Figure 1A**; *P*=0.0191). Subsequently, OS event analysis revealed that miR-125a-5p was significantly differentially expressed between the dead and live groups (**Figure 1B**; *P*= 0.006). Therefore, further investigation on miR-125a-5p expression was deemed necessary. Similarly, no significant differences were found in the clinical stage, histologic grade, pathologic stage, lymph node neck dissection, or lymphovascular invasion (**Figures 1C–G**). In terms of radiation therapy analysis, the statistically significant difference was identified between patients who received radiation therapy (Yes group) and those who did not (No group) (**Figure 1H**; *P*= 0.0466), and the receiver operating characteristic curve determined the AUC value of 0.562 for the diagnosis of radiation therapy (**Figure 1I**).

### 3.2 Determination of the biological function of miR-125a-5p in LSCC

**Figure 2:**
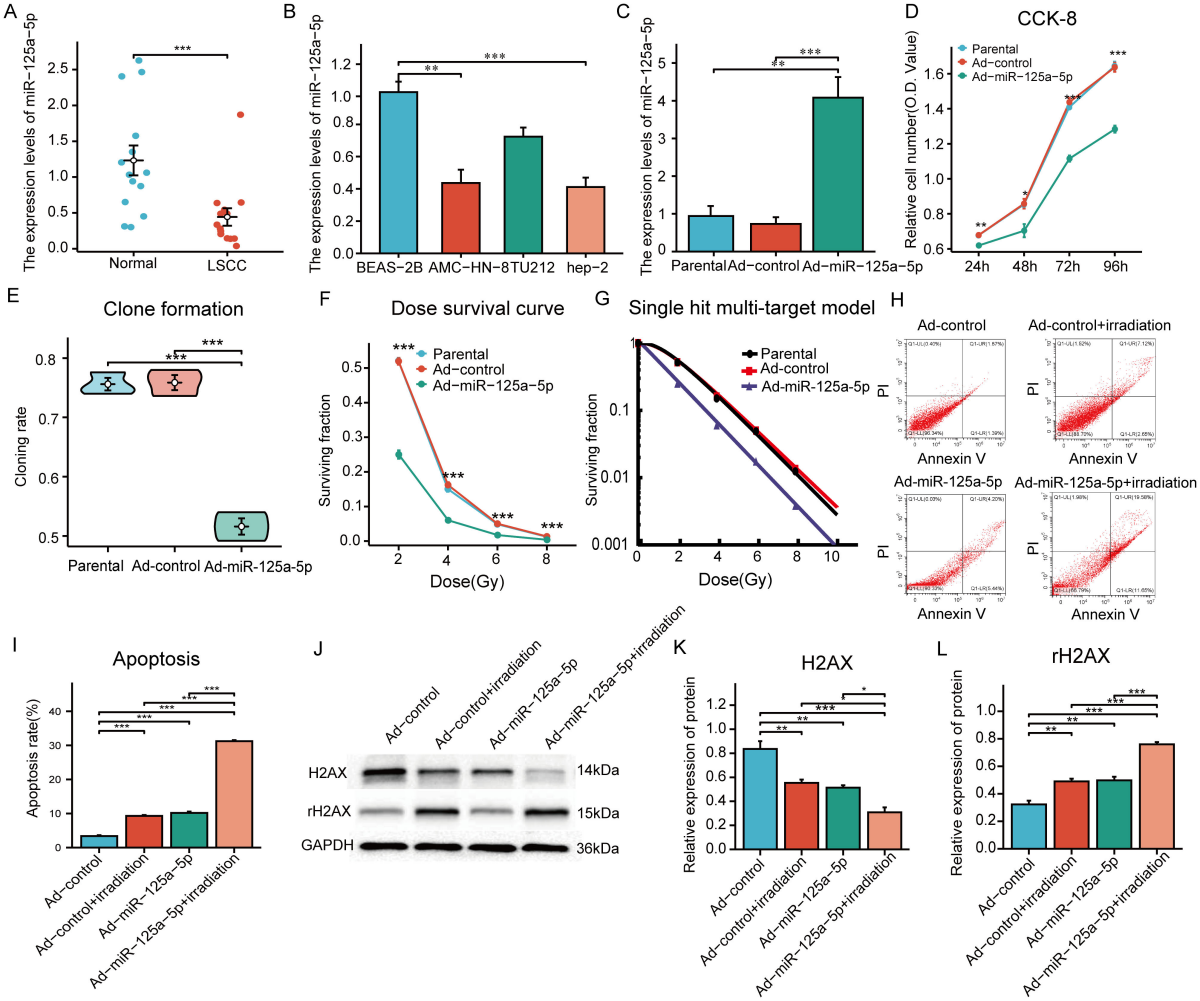
The role and function of miR-125a-5p in LSCC: **(A)** MiR-125a-5p expression in laryngeal cancer tissues and adjacent normal tissues measured using RT-PCR analysis. **(B)** MiR-125a-5p expression in various laryngeal cancer cell lines. **(C)** MiR-125a-5p expression in AMN-HC-8 cells after transfection with miR-125a-5p. **(D)** The CCK-8 assay was used to analyze alterations in the growth potential of laryngeal cancer cells following transfection with miR-125a-5p. **(E)** Furthermore, changes in the ability to form colonies after introducing miR-125a-5p were demonstrated using the colony formation test. **(F)** Examination of survival curves showed the significant decrease in the fraction of surviving cells after transfection with miR-125a-5p. **(G)** In addition, the research employed the single hit multi-target approach to evaluate changes in sensitivity to radiation after introducing miR-l25a-5p through transfection. **(H, I)** Flow cytometry validation demonstrated that transfection with miR-l25a-5p facilitated apoptosis. **(J–L)** Furthermore, the expression levels of DDR pathway-related genes H2AX and rH2AX were assessed using western blotting analysis. (^*^ *P*<0.05, ^**^ *P*<0.01, and ^***^ *P*<0.001).

RT-PCR analysis was conducted on 14 LSCC sample and normal tissue pairs and revealed that miR-125a-5p expression in LSCC was significantly lower than that in adjacent non-cancerous tissues (**Figure 2A**; *P*=0.0008). Consistency was observed between clinical tissue samples and data from TCGA database. Next, we analyzed miR-125a-5p expression levels in laryngeal cancer cells; MiR-125a-5p expression was downregulated in hep-2, TU212, and AMC-HN-8 laryngeal cancer cell lines (**Figure 2B**). Compared to bronchial epithelial cells (BEAS-2B), miR-125a-5p expression in AMC-HN-8 cells was significantly downregulated (*P*=0.0015). The biological role of miR-125a-5p was examined by introducing the overexpression plasmid of miR-125a-5p into AMC-HN-8 cells using lentiviral vectors. Transfection efficiency was assessed by RT-qPCR, which validated the effective upregulation of miR-125a-5p (**Figure 2C**). CCK-8 tests indicated a significant decrease in cell growth rate following the introduction of miR-125a-5p (**Figure 2D**). Additionally, the results of the clone formation tests demonstrated a noticeable decline in cell cloning ability after miR-125a-5p transfection compared to both the parental and Ad-control groups (**Figure 2E**; *P*=0.0004). Upon transfection of miR-125a-5p into LSCC cells, there was a marked decrease in SF in AMC-HN-8 cells with an increasing radiation dose (**Figure 2F**). The radiosensitivity of miR-125a-5p was assessed using a single hit multi-target model (**Figure 2G**). Correspondingly, compared to other groups, the D_0_ and D_37_ values were significantly reduced, indicating an enhancement in radiation resistance. Additionally, SER suggested an improvement in radiosensitivity (**Table 1**). Flow cytometric analysis revealed a significant increase in the death rate of LSCC cells following transfection with miR-125a-5p and subsequent radiotherapy, validating the role of miR-125a-5p in promoting LSCC cell death (**Figures 2H, I**). Before radiotherapy, AMC-HN-8 cells transfected with either Ad-miR-125a-5p or Ad-control showed a significant increase in rH2AX levels and a decrease in H2AX levels. Post-radiotherapy intervention resulted in elevated rH2AX and reduced H2AX levels (**Figures 2J–L**). These results suggest that, following radiotherapy, miR-125a-5p modulates the expression of genes that are involved in the DDR pathway. Hence, our hypothesis was that miR-125a-5p increases sensitivity to radiation by controlling the DDR pathway.

**Table 1:** Single hit multi-target model was used to analyze the effect of miR-125a-5p on the radiosensitivity of laryngeal cancer.

Parameters	Parental	Ad-control	Ad-miR-125a-5p
D_0_(Gy)	1.471	1.546	1.289
D_37_(Gy)	2.762	3.250	2.439
SER	–	–	1.485

### 3.3 Determination of miR-125a-5p target genes and HK2 biological functions

**Figure 3:**
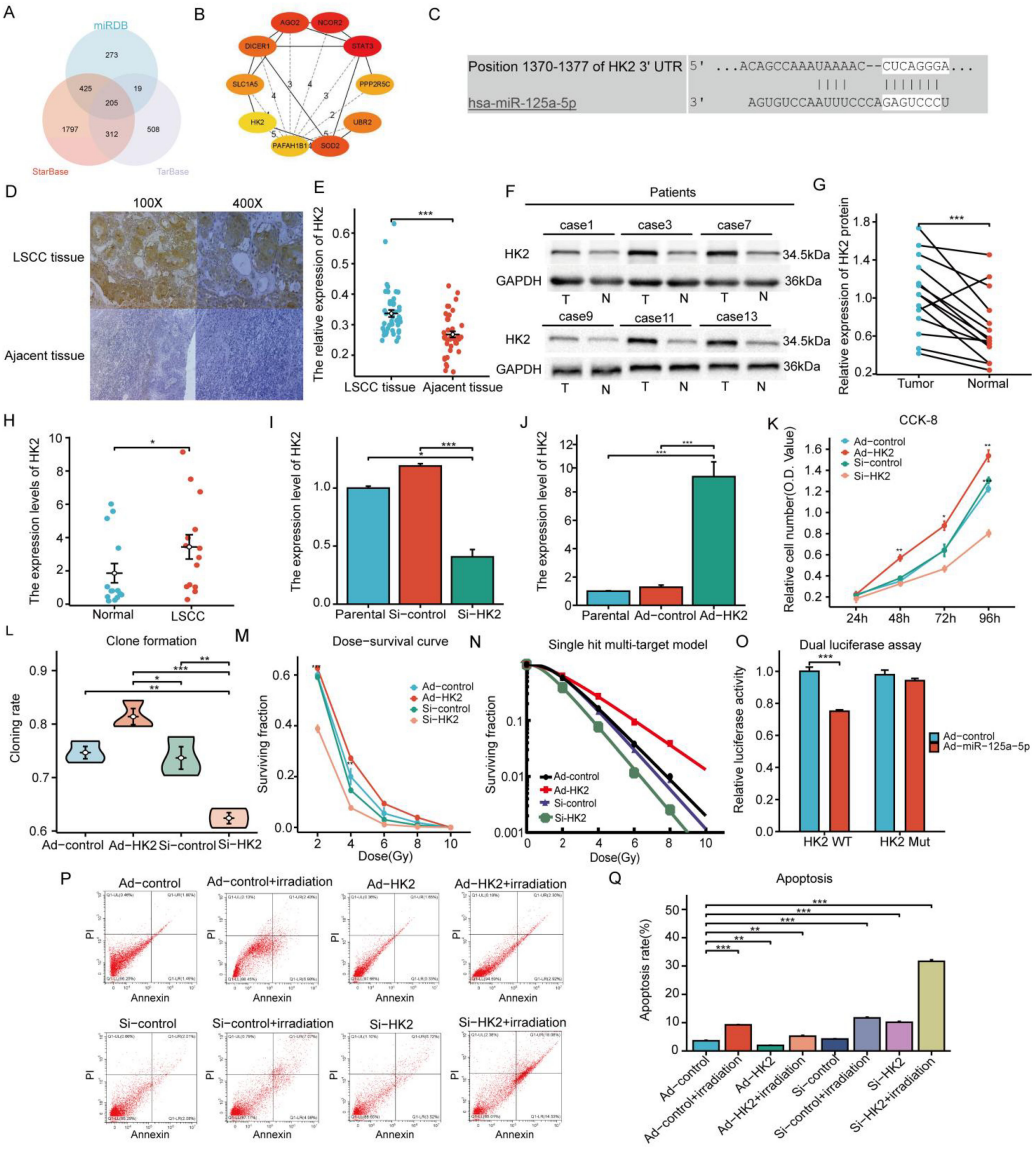
Forecasting the target gene by miR-125a-5p, the expression and biological role of HK2 in LSCC: **(A)** The research involved examining three sets of data, which involved predicting target genes controlled by miR-125a-5p, **(B)** creating the network of PPI and finding the important genes, **(C)** pinpointing the exact location where HK2 and miR-125a-5p bind, and **(D, E)** comparing levels of HK2 in laryngeal cancer tissues to nearby healthy tissues using immunohistochemical analysis. **(F, G)** Western blotting was performed to assess the HK2 expression levels in laryngeal cancer tissues compared to nearby non-cancerous tissues. **(H)** RT-PCR was used to assess HK2 expression levels in laryngeal cancer tissues in comparison to nearby non-cancerous tissues. **(I, J)** Furthermore, RT-PCR was performed to evaluate the effect on HK2 expression levels in AMN-HC-8 cells when either increased or decreased. **(K)** The ability of proliferation in laryngeal cancer cells was evaluated by using the CCK-8 assay after altering the expression levels of HK2. **(L)** The clonogenic assay was employed to investigate alterations in clonability observed in laryngeal cancer cells following modulation of HK2. **(M)** The dose survival curve demonstrated variations in surviving fraction resulting from up-regulation or down-regulation of HK2. **(N)** The comprehensive analysis was conducted using single hit multi-target model to investigate the impact of modulating HK2 expression levels on radiotherapy sensitivity. **(O)** Moreover, the luciferase test was used to verify the specific interaction between miR-125a-5p and HK2. **(P, Q)** Flow cytometry was used to confirm the impact of modified HK2 expression on apoptosis control. (^*^ *P*<0.05, ^**^ *P*<0.01, ^***^ *P*<0.001).

MiR-125a-5p target genes were identified using TarBase v.8, StarBase, and miRDB databases, which yielded predictions for 1044, 2739, and 922 target genes, respectively. A total of 205 target genes were identified by cross-referencing the results from these databases (**Figure 3A**). The key genes of notable significance were identified using the PPI network with the CytoHubba plugin (version 3.8.0). Through an analysis of the relevant literature, HK2 emerged as the primary candidate target gene (**Figure 3B**) (11–13). Using the TargetScan database, we predicted the potential binding sites of miR-125a-5p on HK2 (**Figure 3C**). Tissue immunohistochemistry revealed a notable increase in HK2 expression levels in LSCC tissues compared to the corresponding adjacent non-cancerous tissues (**Figures 3D, E**). This observation was corroborated with western blotting and RT-PCR (**Figures 3F–H**). Lentiviral transfection effectively modulated HK2 expression; HK2 was upregulated in the Ad-HK2 group and downregulated in the Si-HK2 group (**Figures 3I, J**). CCK-8 experimental findings showed that increasing HK2 levels significantly improved cell proliferation, whereas decreasing levels led to a notable decrease in the cellular proliferation rate (**Figure 3K**). Additionally, HK2 upregulation significantly improved clonal ability, whereas its downregulation weakened clonal ability (**Figure 3L**; *P*=0.0033). Following HK2 upregulation, AMC-HN-8 cells were exposed to varying doses of X-rays based on the dose-survival curve, resulting in a significant increase in the surviving fraction compared to the control group. Conversely, the reduction in cell viability was found to be statistically significant upon downregulation of HK2 expression, as compared to the control group(**Figure 3M**). The radiosensitivity of laryngeal cancer cells was assessed using a single hit multi-target model (**Figure 3N**). In the Ad-HK2 group, D_0_ = 1.951 Gy, and D_37_ = 3.422 Gy were higher than those in the control group. The Ad-HK2 cohort (SER=0.838) indicated the decreased sensitivity to radiation. In contrast, the Si-HK2 group exhibited lower D_0_ = 1.146 Gy and D_37_ = 2.224 Gy than the control group, indicating a decrease in resistance to radiation (SER = 1.274) (**Table 2**). Importantly, Si-HK2 displayed a significantly higher radiosensitivity than Ad-HK2. Therefore, the findings suggest that HK2 hinders the sensitivity of laryngeal cancer cells to radiation. Dual luciferase assays validated the notable reduction in luciferase function in the wild-type (HK2 WT) cohort following miR-125a-5p transfection (*P*=0.008), with no substantial impact observed in the mutant (HK2 Mut) cohort (**Figure 3O**; *P*=0.3085). Flow cytometry experiments further validated these results, showing a decrease in the apoptosis rate in the Ad-HK2 groups compared to that in the Ad-control groups following radiotherapy, correlating with an increase in HK2 expression levels (*P*=0.0095). Following a decrease in HK2 expression, the Si-HK2 group showed a significantly increased apoptosis rate compared to the Si-control group after radiotherapy (*P*<0.0001) (**Figures 3P, Q**). These findings indicate that HK2 inhibits laryngeal cancer cell apoptosis.

**Table 2:** Single hit multi-target model was used to analyze the effect of HK2 on the radiosensitivity of laryngeal cancer.

Parameters	Ad-control	Ad-HK2	Si-control	Si-HK2
D_0_(Gy)	1.341	1.951	1.225	1.146
D_37_(Gy)	2.868	3.422	2.832	2.224
SER	-	0.838	-	1.274

### 3.4 The impact of miR-125a-5p-targeted HK2 on LSCC biological processes

**Figure 4:**
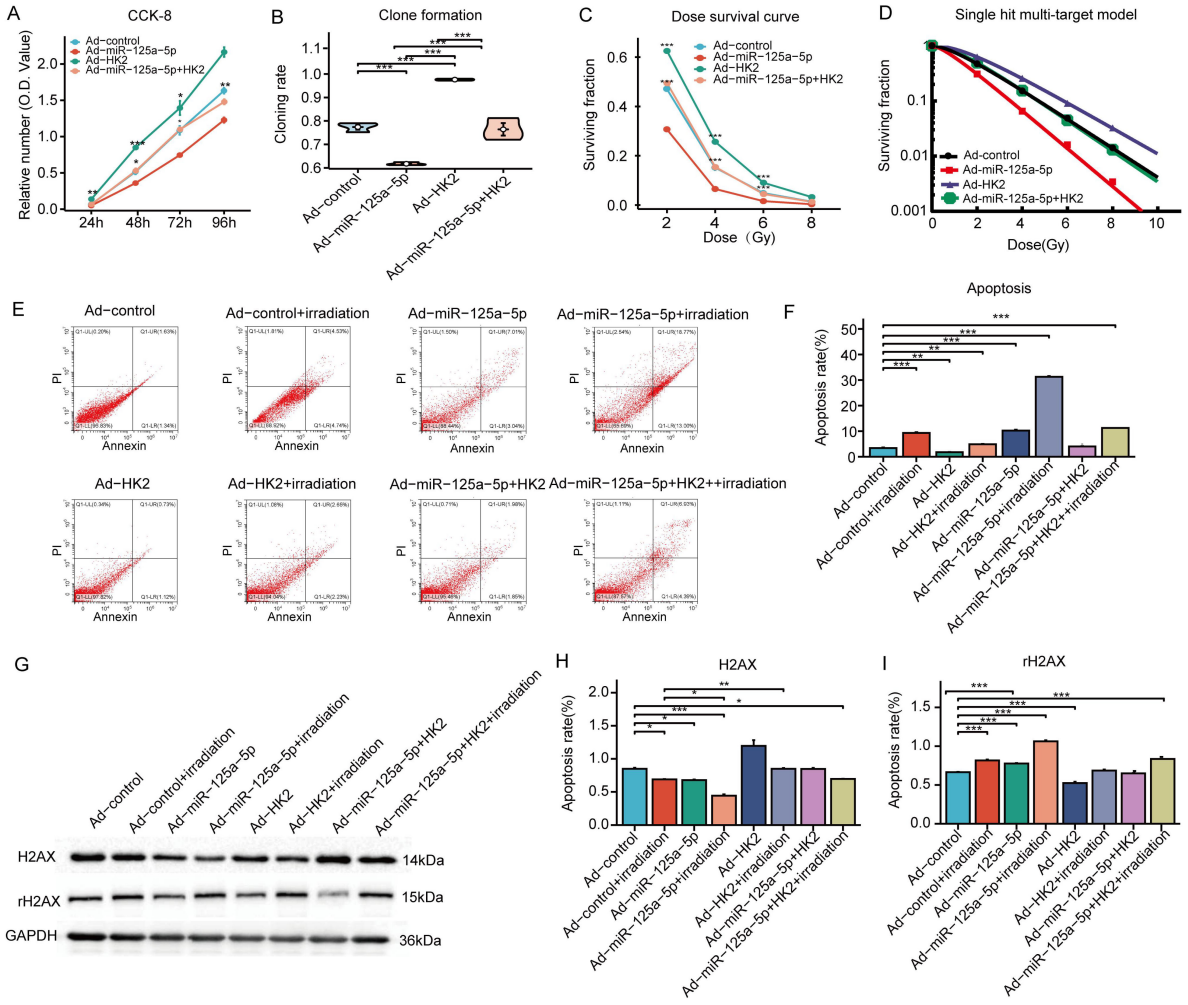
MiR-125a-5p regulates LSCC biological function and radiosensitivity by targeting HK2: **(A)** The regulation of LSCC cell proliferation is specifically mediated by miR-125a-5p through HK2 targeting. **(B)** This targeting of HK2 by miR-125a-5p impacts the clonogenic potential of LSCC cells. **(C)** Additionally, miR-125a-5p plays a role in determining the survival of laryngeal cancer cells under varying doses of irradiation by targeting HK2. **(D)** The single hit multi-target model approach was employed to assess alterations in the radiosensitivity of laryngeal cancer cells. **(E, F)** MiR-125a-5p modulates apoptosis in laryngeal cancer cells by interacting with HK2. **(G–I)** MiR-125a-5p regulates the levels of H2AX and rH2AX by targeting HK2. (^*^ *P*<0.05, ^**^ *P*<0.01, and ^***^ *P*<0.001).

The CCK-8 study revealed significant experimental evidence demonstrating the inhibitory effect of miR-125a-5p on cell growth, whereas increasing HK2 levels enhanced cell proliferation. Reversal of the inhibitory effect on cell growth caused by overexpression of miR-125a-5p was observed upon cotransfection with HK2 and miR-125a-5p (**Figure 4A**). Increasing of miR-125a-5p led to a reduction in clonability, whereas upregulation of HK2 expression enhanced the clonability of AMC-HN-8 cells. It restored clonability to levels observed prior to transfection by co-transfection with both miR-125a-5p and HK2 (**Figure 4B**). Compared to the control group the transfection-mediated introduction of miR-125a-5p resulted in a significant reduction in cell viability following exposure to varying doses of X-ray radiation. Conversely, up-regulation of HK2 resulted in a greater proportion of surviving cells in comparison to the control group. However, co-transfection of miR-125a-5p and HK2 did not lead to a significant alteration in the cell survival rate (**Figure 4C**). Examination of radiosensitivity using a single hit multi-target model showed that the Ad-miR-125a-5p group exhibited the highest D_0_ and D_37_ values, indicating enhanced radiosensitivity resulting from miR-125a-5p overexpression (**Figure 4D**). Conversely, the Ad-HK2 group exhibited lower D_0_ and D_37_ values, suggesting that HK2 induced radioresistance. Examination of SER values indicated that the Ad-miR-125a-5p group had the highest SER and enhanced sensitivity to radiation. Conversely, the Ad-HK2 group showed the lowest SER, indicating a contrasting response to radiotherapy. Cells co-transfected with both miR-125a-5p and HK2 exhibited greater radiation sensitivity than that of the control group (**Table 3**). Flow cytometry revealed a notable increase in apoptosis following AMC-HN-8 cell transfection with miR-125a-5p. Conversely, the increase in HK2 expression led to a reduction in the apoptosis rate; However, this effect was reversed by co-transfection with miR-125a-5p and HK2, with levels comparable to that of the control group (**Figures 4E, F**). Overall, the findings suggest that miR-125a-5p promotes laryngeal cancer cell death and enhances cell sensitivity to radiation. Conversely, HK2 inhibits apoptosis and induces radioresistance. Additionally, miR-125a-5p binding to HK2 modulates laryngeal cancer cell apoptosis. HK2 suppressed the DDR pathway-related gene, rH2AX, while simultaneously increasing H2AX expression in AMC-HN-8 cells,. Conversely, miR-125a-5p inhibited tumor growth by increasing rH2AX levels and decreasing H2AX levels. However, co-transfection with HK2 and miR-125a-p ameliorated separate regulatory effects on genes linked to the DDR pathway, causing insignificant alterations in H2AX and rH2AX expression (**Figures 4G–I**). Finally, miR-125a-5p selectively targeted HK2 and influenced the genes related to the DDR pathway.

**Table 3:** Single hit multi-target model was used to analyze the effect of miR-125a-5p targeting HK2.

Parameters	Ad-control	Ad-miR-125a-5p	Ad-HK2	Ad-miR-125a-5p+HK2
D_0_(Gy)	1.093	0.702	1.478	1.186
D_37_(Gy)	2.737	1.965	3.343	2.744
SER	-	1.393	0.819	0.997

## 4 Discussion

LSCC, the predominant cancer found in the head and neck area, mainly affects individuals between the ages of 55 and 65 years and is on the rise among individuals under 40 years of age (14, 15). Despite advancements in diagnostic tools and treatments for laryngeal cancer, the five-year survival rate for individuals diagnosed with laryngeal cancer remains disappointingly low. Approximately 60% of diagnoses occur in the late stages, leading to a survival rate of less than 50% (16–18). The identification of laryngeal cancer biomarkers can improve LSCC diagnosis, treatment, and patient outcomes via targeted therapies and reduce mortality rates.

MiRNAs have a significant impact on the intricate progression of LSCC. Thus, the identification of new genes linked to radiotherapy response is essential for treating patients with LSCC. Current research has been dedicated to investigating the significance of miRNAs in the progression and management of LSCC. The role of miR-424-5p has been found to be associated with augmenting cellular proliferation and motility in LSCC (19). Sun et al. showed that miR-125a targets HK2, leading to reduced growth and glycolysis and increased apoptosis in LSCC (11). Cancer therapy could potentially benefit from targeting HK2, a vital enzyme involved in aerobic glycolysis that is frequently upregulated in cancer cells. In breast cancer cells, through the mTOR pathway by specifically targeting HK2, miR-216b promotes autophagy and apoptosis (20). HK2 triggers programmed cell death and cellular self-digestion in skin cancer cells (21). The impact of miR-125a-5p on the sensitivity to radiotherapy remains unexplored, despite extensive research into its biological function in different cancer types.

In this investigation, we evaluated the impact of miR-125a-5p and its corresponding target gene HK2 on LSCC while exploring the molecular pathways involved in the response to radiation therapy. RT-PCR analysis revealed a significant reduction in the expression levels of miR-125a-5p in LSCC tissues. On the contrary, there was a significant rise observed in HK2 levels, suggesting a strong negative correlation between miR-125a-5p and HK2 expression. Immunohistochemistry and western blotting examinations revealed elevated HK2 expression in LSCC tissues compared to non-cancerous tissues. Dual-luciferase experiments have provided confirmation that HK2 is the direct target of miR-125a-5p, aligning with the findings of the prior investigation conducted by Sun et al. (11). Increased miR-125a-5p expression reduced LSCC cell growth and clonality and induced cell death. According to the single hit multi-target model, miR-125a-5p enhances the effectiveness of radiation therapy, whereas HK2 reduces the effectiveness of radiation therapy. MiR-125a-5p suppresses LSCC by regulating HK2, which inhibits tumor growth. The biological effects of HK2 were suppressed by co-transfection with miR-125a-5p and HK2.

MiRNAs can enhance the sensitivity of tumors to radiation by controlling various pathways, particularly by influencing the tumor microenvironment, cell cycle checkpoints, DNA repair mechanisms, cell death processes, and signaling pathways related to radiation. By suppressing these pathways, it is feasible to increase tumor radiosensitivity while minimizing its impact on adjacent normal tissues. Tumor cells use DDR pathways to fix radiation-induced DNA damage, prompting the key area of research in radiotherapy (22). Exposure to radiotherapy or ionizing radiation can trigger the DDR in tumors, and the determination of whether cells undergo DNA repair or apoptosis in the presence of substantial DNA damage is governed by a mechanism known as DDR. DDR encompasses various intricate processes, including repair mechanisms such as base excision and the mending of both single-strand and double-strand breaks (23).

There are two primary methods for cancer cells to fix DNA double-strand breaks: the non-homologous end-joining (NHEJ) and homologous recombination (HR) pathways, with NHEJ being particularly important. Enhanced radiosensitivity can be achieved by controlling various molecules, including H2AX, ATM, RAD51, Cdc25A, PLK1, HIF-1, and VEGF, that play the importment role in DNA repair (24, 25). Recent research suggests that miRNAs affect DNA repair proteins and regulate DNA double-strand break repair in tumors by controlling key genes in the DDR pathway. For example, miR-211 enhances platinum sensitivity in ovarian cancer by suppressing DDR, while prostate cancer is associated with the induction of DNA damage by miR-346 (26, 27). Radiation therapy causes DNA damage in cancer cells, leading to the activation of their natural DNA repair mechanism involving the phosphorylation of H2AX into rH2AX, which recruits DNA repair proteins to the damaged site (28). Therefore, rH2AX serves as a key marker for evaluating the progression of DNA repair, with both H2AX and rH2AX playing essential roles in regulating the DDR pathway.

A significant association between HK2 and tumor radiosensitivity has been established. Dogra et al. demonstrated that HK2 influences cancer treatment response by affecting the ATM network-associated DDR pathway, ultimately impacting renal cell carcinoma sensitivity to cisplatin (29). Wu et al. showed that miR-195 increases radiosensitivity in esophageal squamous cells by targeting HK2, leading to enhanced apoptosis after radiotherapy (30). Increased levels of HK2 are closely linked to increased radiation therapy sensitivity in cervical squamous cell carcinoma and can serve as a reliable predictor of this cancer type (31).

This study investigated the impact of miR-125a-5p on apoptosis, DNA damage, and DNA repair in LSCC cells during radiotherapy. The addition of miR-125a-5p after radiotherapy increased apoptosis and double-strand breaks (DSB).We have also observed that miR-125a-5p boosts H2AX phosphorylation and affects the pathways involved in the response to DNA damage. Increased rH2AX in LSCC cells with miR-125a-5p overexpression suggested impaired DSB repair. Increased HK2 levels in LSCC cells decreased rH2AX levels and prevented cell death; However, reintroduction of miR-125a-5p through cotransfection led to the recovery of rH2AX expression. Both miR-125a-5p and HK2 play key roles in LSCC pathogenesis via DDR pathway modulation, hindering repair mechanisms and increasing radiosensitivity.

In summary, through extensive testing, we discovered that miR-125a-5p targets HK2, affects gene expression in the DDR pathway, targeting miR-125a-5p has been demonstrated to augment the radiosensitivity of LSCC cells, thereby potentially ameliorating the therapeutic efficacy in patients afflicted with laryngeal cancer. These findings hold substantial implications for optimizing radiotherapy effectiveness and identifying prospective biomarkers for future interventions.

## 5 Conclusion

The inhibitory effects of miR-125a-5p on cell proliferation, as well as its capacity to enhance cell death and increase sensitivity towards radiation treatment, impede tumor growth in LSCC, while HK2 could result in the promotion of cellular growth, prevention of apoptosis, and a decrease in sensitivity towards radiation. MiR-125a-5p targets HK2 and affects sensitivity to radiation by influencing DDR pathway-associated genes in LSCC. This discovery suggests a potential strategy for improving the effectiveness of radiotherapy in the treatment of LSCC and establishes a foundation for future investigations into the modulation of radiosensitivity.

## Data Availability

The original contributions presented in the study are included in the article/**Supplementary Material**. Further inquiries can be directed to the corresponding authors.
