# Supplementary material for: MiR-125a-5p regulates the radiosensitivity of laryngeal squamous cell carcinoma via HK2 targeting through the DDR pathway
Source: Front Oncol. 2024 Aug 19;14:1438722. doi: 10.3389/fonc.2024.1438722 (PMC11366599; doi:10.3389/fonc.2024.1438722)
Supplement: Supplementary file 1 [file Datasheet1.pdf]

The expression levels				
id	group	Mean	SD	P
hsa-miR-125a-3p	Normal	2.5914	0.61474	0.0467
hsa-miR-125a-3p	Tumor	2.8597	0.67719	
hsa-miR-125a-5p	Normal	7.9257	0.7667	0.0191
hsa-miR-125a-5p	Tumor	7.6544	0.81783	
hsa-miR-125b-1-3p	Normal	1.6325	0.57376	0.0011
hsa-miR-125b-1-3p	Tumor	1.2687	0.61416	
hsa-miR-125b-2-3p	Normal	5.785	0.71383	1.69e-09
hsa-miR-125b-2-3p	Tumor	4.32	1.1931	
hsa-miR-125b-5p	Normal	9.5765	0.52961	7.6e-11
hsa-miR-125b-5p	Tumor	8.464	0.67772	
OS event				
id	group	Mean	SD	P
hsa-miR-125a-3p	Normal	2.6428	0.79099	0.1824
hsa-miR-125a-3p	Tumor	2.7297	0.78237	
hsa-miR-125a-5p	Normal	8.2609	0.8367	0.0060
hsa-miR-125a-5p	Tumor	8.0586	0.86888	
hsa-miR-125b-1-3p	Normal	1.153	0.66746	0.1031
hsa-miR-125b-1-3p	Tumor	1.2113	0.57368	
hsa-miR-125b-2-3p	Normal	4.1396	1.5258	0.0127
hsa-miR-125b-2-3p	Tumor	3.7989	1.3709	
hsa-miR-125b-5p	Normal	8.6971	0.96078	0.0305
hsa-miR-125b-5p	Tumor	8.5011	0.86605	

Clinical stage		
group	Mean	SD
Normal	7.959	0.78929
Stage I	8.1751	0.76016
Stage II	8.2722	0.90067
Stage III	7.9363	0.87769
Stage IV	8.1988	0.82626
Group I	Group J	P
Normal	Stage I	0.8709
Normal	Stage II	0.2481
Normal	Stage III	0.9999
Normal	Stage IV	0.4018
Stage I	Stage II	0.9893
Stage I	Stage III	0.7614
Stage I	Stage IV	0.9999
Stage II	Stage III	0.0393
Stage II	Stage IV	0.9468
Stage III	Stage IV	0.0515
Histologic grade		
group	Mean	SD
Normal	7.959	0.78929
G1	8.1753	0.79294
G2	8.1217	0.83557
G3	8.2886	0.89408
G4	8.7218	0.20837
Group I	Group J	P

Normal	G1	1
Normal	G2	1
Normal	G3	0.1772
Normal	G4	0.0792
G1	G2	1
G1	G3	1
G1	G4	0.489
G2	G3	0.8306
G2	G4	0.2639
G3	G4	0.869
Patheologic stage		
group	Mean	SD
Normal	7.959	0.78929
Stage I	8.0653	0.93751
Stage II	8.1928	0.90882
Stage III	8.196	0.87737
Stage IV	8.1556	0.8499
Group I	Group J	P
Normal	Stage I	0.9870
Normal	Stage II	0.6152
Normal	Stage III	0.5891
Normal	Stage IV	0.6267
Stage I	Stage II	0.9655
Stage I	Stage III	0.9609
Stage I	Stage IV	0.9855
Stage II	Stage III	1.0000
Stage II	Stage IV	0.9975
Stage III	Stage IV	0.9961
Lymphnode neck dissection		
group	Mean	SD
Normal	7.959	0.78929
No	8.1334	0.89989
Yes	8.1904	0.84264
Group I	Group J	P
Normal	No	0.4235
Normal	Yes	0.1583
No	Yes	1
Lymphovascular invasion		
group	Mean	SD
Yes	8.274	0.89829
No	8.1833	0.83686
Group I	Group J	P
Yes	No	0.2325
Radiation therapy		
group	Mean	SD
Yes	8.2555	0.85004
No	8.0886	0.86709
Group I	Group J	P
Yes	No	0.0466
Tissure PCR		
group	Mean	SD
normal	1.2327	0.7804

LSCC	0.4435	0.45583
Group I	Group J	P
normal	LSCC	0.0008
Cell PCR		
group	Mean	SD
BEAS-2B	1	0.27327
AMC-HN-8	0.43654	0.24582
TU212	0.72392	0.16865
hep-2	0.4107	0.17711
Group I	Group J	P
BEAS-2B	AMC-HN-8	0.0015
BEAS-2B	TU212	4.41E-01
BEAS-2B	hep-2	4.00E-04
AMC-HN-8	TU212	0.3611
AMC-HN-8	hep-2	1
TU212	hep-2	0.17
Transfection		
group	Mean	SD
Parental	1	0.61771
Ad-control	0.73013	0.53993
Ad-miR-125a-5p	4.0812	1.6413
Group I	Group J	P
Parental	Ad-control	1
Parental	Ad-miR-125a-5p	0.0021
Ad-control	Ad-miR-125a-5p	0.0004

miR-125a-5p CCK-8			
Time	Group	Mean	SD
24h	Parental	0.67733	0.018448
24h	Ad-control	0.67867	0.013577
24h	Ad-miR-125a-5p	0.61933	0.0075056
48h	Parental	0.85333	0.025716
48h	Ad-control	0.85767	0.047721
48h	Ad-miR-125a-5p	0.70367	0.064694
72h	Parental	1.4083	0.014844
72h	Ad-control	1.4377	0.041861
72h	Ad-miR-125a-5p	1.1157	0.030288
96h	Parental	1.6437	0.043662
96h	Ad-control	1.637	0.045431
96h	Ad-miR-125a-5p	1.2837	0.034775
Time	Group I	Group J	P
24h	Ad-control	Parental	1
24h	Ad-miR-125a-5p	Parental	0.0066
24h	Ad-miR-125a-5p	Ad-control	0.0059
48h	Ad-control	Parental	1
48h	Ad-miR-125a-5p	Parental	0.0281
48h	Ad-miR-125a-5p	Ad-control	0.0248
72h	Ad-control	Parental	0.8732
72h	Ad-miR-125a-5p	Parental	7.60E-05
72h	Ad-miR-125a-5p	Ad-control	4.37E-05
96h	Ad-control	Parental	1

96h	Ad-miR-125a-5p	Parental	0.0001
96h	Ad-miR-125a-5p	Ad-control	0.0001

miR-125a-5p cloning rate		
Group	Mean	SD
Parental	0.756	0.01833
Ad-control	0.75867	0.02203
Ad-miR-125a-5p	0.516	0.024
Group I	Group J	P
Parental	Ad-control	0.9875
Parental	Ad-miR-125a-5p	2.38e-05
Ad-control	Ad-miR-125a-5p	2.23e-05

MiR-125a-5p SF			
Group 1	Group 2	Mean	SD
2Gy	Parental	0.52054	0.015737
2Gy	Ad-control	0.51966	0.017242
2Gy	Ad-miR-125a-5p	0.30638	0.029631
4Gy	Parental	0.15057	0.013596
4Gy	Ad-control	0.16211	0.014192
4Gy	Ad-miR-125a-5p	0.073675	0.0013443
6Gy	Parental	0.048772	0.003345
6Gy	Ad-control	0.050263	0.003568
6Gy	Ad-miR-125a-5p	0.021365	0.0022978
8Gy	Parental	0.012422	0.001321
8Gy	Ad-control	0.013471	0.001229
8Gy	Ad-miR-125a-5p	0.0046941	0.00035062
Group 1	Group 2	p	
2Gy	Parental	0.9625	
2Gy	Ad-control	1.99E-05	
2Gy	Ad-miR-125a-5p	2.04E-05	
4Gy	Parental	0.2603	
4Gy	Ad-control	0.0002	
4Gy	Ad-miR-125a-5p	7.65E-05	
6Gy	Parental	0.5798	
6Gy	Ad-control	3.81E-05	
6Gy	Ad-miR-125a-5p	2.81E-05	
8Gy	Parental	0.2716	
8Gy	Ad-control	0.0001	
8Gy	Ad-miR-125a-5p	5.38E-05	

miR-125a-5p apoptosis		
Group	Mean	SD
Ad-control	3.3633	0.45391
Ad-control+irradiation	9.2967	0.46058
Ad-miR-125a-5p	10.2	0.64815
Ad-miR-125a-5p+irradiation	31.227	0.54501
Group I	Group J	p
Ad-control	Ad-control+irradiation	3.83E-06
Ad-control	Ad-miR-125a-5p	1.30E-06
Ad-control	Ad-miR-125a-5p+irradiation	1.61E-12

Ad-control+irradiation	Ad-miR-125a-5p	0.2389
Ad-control+irradiation	Ad-miR-125a-5p+irradiation	1.17E-10
Ad-miR-125a-5p	Ad-miR-125a-5p+irradiation	2.07E-10
miR-125a-5p WB H2AX		
Group	Mean	SD
Ad-control	0.83558	0.11114
Ad-control+irradiation	0.5537	0.046523
Ad-miR-125a-5p	0.51393	0.033494
Ad-miR-125a-5p+irrdiation	0.30927	0.070345
Group I	Group J	p
Ad-control	Ad-control+irradiation	0.0058
Ad-control	Ad-miR-125a-5p	0.0026
Ad-control	Ad-miR-125a-5p+irrdiation	8.70E-05
Ad-control+irradiation	Ad-miR-125a-5p	0.9023
Ad-control+irradiation	Ad-miR-125a-5p+irrdiation	0.0132
Ad-miR-125a-5p	Ad-miR-125a-5p+irrdiation	0.0333
miR-125a-5p WB rH2AX		
Group	Mean	SD
Ad-control	0.32208	0.046952
Ad-control+irradiation	0.48995	0.033997
Ad-miR-125a-5p	0.49734	0.045062
Ad-miR-125a-5p+irrdiation	0.75943	0.028692
Group I	Group J	p
Ad-control	Ad-control+irradiation	0.0036
Ad-control	Ad-miR-125a-5p	0.0027
Ad-control	Ad-miR-125a-5p+irrdiation	3.94E-06
Ad-control+irradiation	Ad-miR-125a-5p	0.9954
Ad-control+irradiation	Ad-miR-125a-5p+irrdiation	0.0001
Ad-miR-125a-5p	Ad-miR-125a-5p+irrdiation	0.0002

HK2 IHC		
Group	mean	SD
cancer	0.336764663	0.077398305
adjacent tissue	0.268010576	0.06499681
Group I	Group J	p
cancer	adjacent tissue	6.99E-06
HK2 Tissue WB		
Group	mean	SD
Tumor	1.0366	0.39407
Normal	0.68706	0.36199
Group I	Group J	p
Tumor	Normal	0.0009
HK2 tissure PCR		
Group	mean	SD
normal	1.862735208	2.164737214
LSCC	3.441589264	2.72911032
Group I	Group J	p
normal	LSCC	0.0444
HK2 cell PCR		
Group	mean	SD
Parental	1	0.048837
Si-control	1.1902	0.061299

Si-HK2	0.40754	0.18921
Group I	Group J	p
Parental	Si-control	0.0569
Parental	Si-HK2	0.0447
Si-control	Si-HK2	5.23E-06
Group	mean	SD
Parental	1	0.088879
Ad-control	1.2756	0.44646
Ad-HK2	9.0798	3.1407
Group I	Group J	p
Parental	Ad-control	0.2209
Parental	Ad-HK2	0.0001
Ad-control	Ad-HK2	0.0002

HK2 CCK8			
Group 1	Group 2	Mean	SD
24h	Ad-control	0.22367	0.035247
24h	Ad-HK2	0.22433	0.045633
24h	Si-control	0.21367	0.053163
24h	Si-HK2	0.18367	0.060682
48h	Ad-control	0.34867	0.05058
48h	Ad-HK2	0.57067	0.050964
48h	Si-control	0.38	0.025357
48h	Si-HK2	0.32533	0.038109
72h	Ad-control	0.64533	0.084878
72h	Ad-HK2	0.87567	0.075791
72h	Si-control	0.64167	0.10055
72h	Si-HK2	0.46733	0.048789
96h	Ad-control	1.2253	0.049501
96h	Ad-HK2	1.5383	0.095133
96h	Si-control	1.3057	0.050856
96h	Si-HK2	0.80333	0.05428
Time	Group I	GroupJ	p
24h	Ad-HK2	Ad-control	0.9873
24h	Si-control	Ad-control	0.8111
24h	Si-HK2	Ad-control	0.3521
24h	Si-control	Ad-HK2	0.7988
24h	Si-HK2	Ad-HK2	0.3445
24h	Si-HK2	Si-control	0.4798
48h	Ad-HK2	Ad-control	0.0002
48h	Si-control	Ad-control	0.3937
48h	Si-HK2	Ad-control	0.521
48h	Si-control	Ad-HK2	0.0006
48h	Si-HK2	Ad-HK2	0.0001
48h	Si-HK2	Si-control	0.1545
72h	Ad-HK2	Ad-control	0.0076
72h	Si-control	Ad-control	0.9565
72h	Si-HK2	Ad-control	0.0257
72h	Si-control	Ad-HK2	0.007
72h	Si-HK2	Ad-HK2	0.0002
72h	Si-HK2	Si-control	0.028
96h	Ad-HK2	Ad-control	0.0004

96h	Si-control	Ad-control	0.1701
96h	Si-HK2	Ad-control	4.69E-05
96h	Si-control	Ad-HK2	0.0024
96h	Si-HK2	Ad-HK2	7.36E-07
96h	Si-HK2	Si-control	1.32E-05

HK2 cloning rate			
Group	mean	SD	
Ad-control	0.74667	0.020133	
Ad-HK2	0.814	0.026153	
Si-control	0.73667	0.03646	
Si-HK2	0.624	0.018	
Group I	Group J	p	
Ad-control	Ad-HK2	0.0539	
Ad-control	Si-control	0.9641	
Ad-control	Si-HK2	0.0019	
Ad-HK2	Si-control	0.028	
Ad-HK2	Si-HK2	9.43E-05	
Si-control	Si-HK2	0.0033	
HK2 SF			
Dose	Group	Mean	SD
2Gy	Ad-control	0.59908	0.021501
2Gy	Ad-HK2	0.62659	0.0039393
2Gy	Si-control	0.5911	0.0059821
2Gy	Si-HK2	0.38853	0.024759
4Gy	Ad-control	0.19989	0.053397
4Gy	Ad-HK2	0.27209	0.011223
4Gy	Si-control	0.14606	0.0029704
4Gy	Si-HK2	0.077008	0.01318
6Gy	Ad-control	0.05563	0.035861
6Gy	Ad-HK2	0.093964	0.0009236
6Gy	Si-control	0.030351	0.0031254
6Gy	Si-HK2	0.011633	0.00099515
8Gy	Ad-control	0.018727	0.014972
8Gy	Ad-HK2	0.03896	0.0028258
8Gy	Si-control	0.0088458	0.0008672
8Gy	Si-HK2	0.0025446	0.00037658
10Gy	Ad-control	0	0
10Gy	Ad-HK2	0	0
10Gy	Si-control	0	0
10Gy	Si-HK2	0	0
Dose	Group I	Group J	P
2Gy	Ad-HK2	Ad-control	0.4775
2Gy	Si-control	Ad-control	1
2Gy	Si-HK2	Ad-control	1.92E-06
2Gy	Si-control	Ad-HK2	0.1928
2Gy	Si-HK2	Ad-HK2	7.37E-07
2Gy	Si-HK2	Si-control	2.59E-06
4Gy	Ad-HK2	Ad-control	0.0821
4Gy	Si-control	Ad-control	0.2819
4Gy	Si-HK2	Ad-control	0.0041

4Gy	Si-control	Ad-HK2	0.0035
4Gy	Si-HK2	Ad-HK2	0.0002
4Gy	Si-HK2	Si-control	0.101
6Gy	Ad-HK2	Ad-control	0.1877
6Gy	Si-control	Ad-control	0.7436
6Gy	Si-HK2	Ad-control	0.1037
6Gy	Si-control	Ad-HK2	0.0152
6Gy	Si-HK2	Ad-HK2	0.0031
6Gy	Si-HK2	Si-control	1
8Gy	Ad-HK2	Ad-control	0.0706
8Gy	Si-control	Ad-control	0.909
8Gy	Si-HK2	Ad-control	0.1907
8Gy	Si-control	Ad-HK2	0.0078
8Gy	Si-HK2	Ad-HK2	0.0023
8Gy	Si-HK2	Si-control	1
Luciferase assay			
Group1	Group 2	Mean	SD
HK2 WT	Ad-control	1	0.044507
HK2 WT	Ad-miR-125a-5p	0.75096	0.014821
HK2 Mut	Ad-control	0.97897	0.050069
HK2 Mut	Ad-miR-125a-5p	0.94143	0.024619
Group	Group I	Group J	p
HK2 WT	Ad-control	Ad-miR-125a-5p	0.0008
HK2 Mut	Ad-control	Ad-miR-125a-5p	0.3085

HK2 Apoptosis		
Group	Mean	SD
Ad-control	3.58	0.34395
Ad-control+irradiation	9.24	0.17
Ad-HK2	1.9533	0.083267
Ad-HK2+irradiation	5.2333	0.45015
Si-control	4.22	0.14731
Si-control+irradiation	11.693	0.42028
Si-HK2	10.14	0.55678
Si-HK2+irradiation	31.663	0.91221
Group I	Group J	p
Ad-control	Ad-control+irradiation	1.72E-09
Ad-control	Ad-HK2	0.0095
Ad-control	Ad-HK2+irradiation	0.0083
Ad-control	Si-control	0.6865
Ad-control	Si-control+irradiation	8.64E-12
Ad-control	Si-HK2	1.71E-10
Ad-control	Si-HK2+irradiation	2.26E-14
Ad-control+irradiation	Ad-HK2	3.68E-11
Ad-control+irradiation	Ad-HK2+irradiation	2.61E-07
Ad-control+irradiation	Si-control	1.03E-08
Ad-control+irradiation	Si-control+irradiation	0.0001
Ad-control+irradiation	Si-HK2	0.3071
Ad-control+irradiation	Si-HK2+irradiation	2.26E-14
Ad-HK2	Ad-HK2+irradiation	4.01E-06
Ad-HK2	Si-control	0.0004

Ad-HK2	Si-control+irradiation	4.75E-13
Ad-HK2	Si-HK2	7.62E-12
Ad-HK2	Si-HK2+irradiation	2.26E-14
Ad-HK2+irradiation	Si-control	0.1936
Ad-HK2+irradiation	Si-control+irradiation	2.17E-10
Ad-HK2+irradiation	Si-HK2	1.44E-08
Ad-HK2+irradiation	Si-HK2+irradiation	2.26E-14
Si-control	Si-control+irradiation	2.61E-11
Si-control	Si-HK2	8.56E-10
Si-control	Si-HK2+irradiation	2.26E-14
Si-control+irradiation	Si-HK2	0.0139
Si-control+irradiation	Si-HK2+irradiation	2.26E-14
Si-HK2	Si-HK2+irradiation	2.26E-14

miR-125a-5p-HK2 CCK8			
TIime	Group	Mean	SD
24h	Ad-control	0.067035	0.005613
24h	Ad-miR-125a-5p	0.050287	0.0064072
24h	Ad-HK2	0.13964	0.033589
24h	Ad-miR-125a-5p+HK2	0.067183	0.0062666
48h	Ad-control	0.51523	0.058221
48h	Ad-miR-125a-5p	0.35985	0.0069269
48h	Ad-HK2	0.84909	0.028276
48h	Ad-miR-125a-5p+HK2	0.52811	0.056709
72h	Ad-control	1.0854	0.103
72h	Ad-miR-125a-5p	0.74562	0.02475
72h	Ad-HK2	1.3939	0.16854
72h	Ad-miR-125a-5p+HK2	1.0984	0.051662
96h	Ad-control	1.6322	0.067934
96h	Ad-miR-125a-5p	1.2281	0.060835
96h	Ad-HK2	2.1638	0.1135
96h	Ad-miR-125a-5p+HK2	1.4788	0.059611
Time	Group	p	Corrected p
24h	Ad-miR-125a-5p	0.2776	1
24h	Ad-HK2	0.001	0.0059
24h	Ad-miR-125a-5p+HK2	0.992	1
24h	Ad-HK2	0.0003	0.0015
24h	Ad-miR-125a-5p+HK2	0.2737	1
24h	Ad-miR-125a-5p+HK2	0.001	0.006
48h	Ad-miR-125a-5p	0.0023	0.0136
48h	Ad-HK2	1.27E-05	7.62E-05
48h	Ad-miR-125a-5p+HK2	0.7242	1
48h	Ad-HK2	7.02E-07	4.21E-06
48h	Ad-miR-125a-5p+HK2	0.0014	0.0084
48h	Ad-miR-125a-5p+HK2	1.70E-05	0.0001
72h	Ad-miR-125a-5p	0.0037	0.0222
72h	Ad-HK2	0.0063	0.0376
72h	Ad-miR-125a-5p+HK2	0.8808	1
72h	Ad-HK2	5.63E-05	0.0003
72h	Ad-miR-125a-5p+HK2	3.00E-03	0.0179
72h	Ad-miR-125a-5p+HK2	0.0079	0.0471
96h	Ad-miR-125a-5p	0.0002	0.0014

96h	Ad-HK2	3.41E-05	0.0002
96h	Ad-miR-125a-5p+HK2	0.044	0.2642
96h	Ad-HK2	4.83E-07	2.90E-06
96h	Ad-miR-125a-5p+HK2	0.0045	0.0271
96h	Ad-miR-125a-5p+HK2	5.24E-06	3.14E-05

miR-125a-5p-HK2 cloning rate			
Group	Mean	SD	
Ad-control	0.774	0.019698	
Ad-miR-125a-5p	0.61733	0.0061101	
Ad-HK2	0.976	0.004	
Ad-miR-125a-5p+HK2	0.764	0.04613	
GroupI	GroupJ	p	
Ad-control	Ad-miR-125a-5p	0.0003	
Ad-control	Ad-HK2	4.73E-05	
Ad-control	Ad-miR-125a-5p+HK2	0.9607	
Ad-miR-125a-5p	Ad-HK2	5.86E-07	
Ad-miR-125a-5p	Ad-miR-125a-5p+HK2	0.0005	
Ad-HK2	Ad-miR-125a-5p+HK2	3.30E-05	
miR-125a-5p-HK2 SF			
Group 1	Group 2	Mean	SD
2Gy	Ad-control	0.4714	0.015582
2Gy	Ad-miR-125a-5p	0.3073	0.010856
2Gy	Ad-HK2	0.62501	0.0048765
2Gy	Ad-miR-125a-5p+HK2	0.49414	0.0061793
4Gy	Ad-control	0.15246	0.017189
4Gy	Ad-miR-125a-5p	0.065337	0.00042192
4Gy	Ad-HK2	0.25668	0.00675
4Gy	Ad-miR-125a-5p+HK2	0.15411	0.0059882
6Gy	Ad-control	0.048831	0.0038637
6Gy	Ad-miR-125a-5p	0.016048	0.0024798
6Gy	Ad-HK2	0.09066	0.0072498
6Gy	Ad-miR-125a-5p+HK2	0.044776	0.0081061
8Gy	Ad-control	0.014127	0.001862
8Gy	Ad-miR-125a-5p	0.0034398	0.00037128
8Gy	Ad-HK2	0.032398	0.0023391
8Gy	Ad-miR-125a-5p+HK2	0.01313	0.00058332
Dose	Group I	Group J	p
2Gy	Ad-miR-125a-5p	Ad-control	2.92E-07
2Gy	Ad-HK2	Ad-control	4.90E-07
2Gy	Ad-miR-125a-5p+HK2	Ad-control	0.16
2Gy	Ad-HK2	Ad-miR-125a-5p	1.56E-09
2Gy	Ad-miR-125a-5p+HK2	Ad-miR-125a-5p	1.05E-07
2Gy	Ad-miR-125a-5p+HK2	Ad-HK2	1.71E-06
4Gy	Ad-miR-125a-5p	Ad-control	2.51E-05
4Gy	Ad-HK2	Ad-control	6.40E-06
4Gy	Ad-miR-125a-5p+HK2	Ad-control	1
4Gy	Ad-HK2	Ad-miR-125a-5p	5.55E-08
4Gy	Ad-miR-125a-5p+HK2	Ad-miR-125a-5p	2.17E-05
4Gy	Ad-miR-125a-5p+HK2	Ad-HK2	7.23E-06
6Gy	Ad-miR-125a-5p	Ad-control	0.0008

6Gy	Ad-HK2	Ad-control	0.0001
6Gy	Ad-miR-125a-5p+HK2	Ad-control	1
6Gy	Ad-HK2	Ad-miR-125a-5p	1.81E-06
6Gy	Ad-miR-125a-5p+HK2	Ad-miR-125a-5p	0.002
6Gy	Ad-miR-125a-5p+HK2	Ad-HK2	7.34E-05
8Gy	Ad-miR-125a-5p	Ad-control	0.0002
8Gy	Ad-HK2	Ad-control	2.87E-06
8Gy	Ad-miR-125a-5p+HK2	Ad-control	1
8Gy	Ad-HK2	Ad-miR-125a-5p	7.81E-08
8Gy	Ad-miR-125a-5p+HK2	Ad-miR-125a-5p	0.0003
8Gy	Ad-miR-125a-5p+HK2	Ad-HK2	1.90E-06

MiR-125a-5p-HK2 apoptosis		
Group	Mean	SD
Ad-control	3.3633	0.45391
Ad-control+irradiation	9.2967	0.46058
Ad -HK2	1.7633	0.12503
Ad-HK2+irradiation	4.8633	0.25541
Ad-miR-125a-5p	10.2	0.64815
Ad-miR-125a-5p+irradiation	31.227	0.54501
Ad-miR-125a-5p+HK2	4.0133	0.17559
Ad-miR-125a-5p+HK2++irradiation	11.26	0.41328
GroupI	GroupJ	p
Ad-control	Ad-control+irradiation	2.01E-10
Ad-control	Ad -HK2	0.005
Ad-control	Ad-HK2+irradiation	0.0088
Ad-control	Ad-miR-125a-5p	2.58E-11
Ad-control	Ad-miR-125a-5p+irradiation	2.26E-14
Ad-control	Ad-miR-125a-5p+HK2	0.5747
Ad-control	Ad-miR-125a-5p+HK2++irradiation	3.50E-12
Ad-control+irradiation	Ad -HK2	6.94E-12
Ad-control+irradiation	Ad-HK2+irradiation	1.70E-08
Ad-control+irradiation	Ad-miR-125a-5p	0.2153
Ad-control+irradiation	Ad-miR-125a-5p+irradiation	2.26E-14
Ad-control+irradiation	Ad-miR-125a-5p+HK2	1.24E-09
Ad-control+irradiation	Ad-miR-125a-5p+HK2++irradiation	0.0007
Ad -HK2	Ad-HK2+irradiation	2.57E-06
Ad -HK2	Ad-miR-125a-5p	1.22E-12
Ad -HK2	Ad-miR-125a-5p+irradiation	2.26E-14
Ad -HK2	Ad-miR-125a-5p+HK2	0.0001
Ad -HK2	Ad-miR-125a-5p+HK2++irradiation	1.45E-13
Ad-HK2+irradiation	Ad-miR-125a-5p	1.06E-09
Ad-HK2+irradiation	Ad-miR-125a-5p+irradiation	2.26E-14
Ad-HK2+irradiation	Ad-miR-125a-5p+HK2	0.2732
Ad-HK2+irradiation	Ad-miR-125a-5p+HK2++irradiation	6.48E-11
Ad-miR-125a-5p	Ad-miR-125a-5p+irradiation	2.26E-14
Ad-miR-125a-5p	Ad-miR-125a-5p+HK2	1.06E-10
Ad-miR-125a-5p	Ad-miR-125a-5p+HK2++irradiation	0.0998
Ad-miR-125a-5p+irradiation	Ad-miR-125a-5p+HK2	2.26E-14
Ad-miR-125a-5p+irradiation	Ad-miR-125a-5p+HK2++irradiation	2.26E-14
Ad-miR-125a-5p+HK2	Ad-miR-125a-5p+HK2++irradiation	1.18E-11
MiR-125a-5p-HK2 WB H2AX		

Group	Mean	SD
Ad-control	0.85081	0.026489
Ad-control+irradiation	0.68934	0.0093739
Ad-miR-125a-5p	0.6794	0.013259
Ad-miR-125a-5p+irradiation	0.44314	0.03414
Ad-HK2	1.1966	0.15081
Ad-HK2+irradiation	0.8508	0.016001
Ad-miR-125a-5p+HK2	0.84737	0.028121
Ad-miR-125a-5p+HK2+irradiation	0.6956	0.0046332
GroupI	GroupJ	p
Ad-control	Ad-control+irradiation	0.024
Ad-control	Ad-miR-125a-5p	0.0142
Ad-control	Ad-miR-125a-5p+irradiation	0.0009
Ad-control	Ad-HK2	0.2281
Ad-control	Ad-HK2+irradiation	1
Ad-control	Ad-miR-125a-5p+HK2	1
Ad-control	Ad-miR-125a-5p+HK2+irradiation	0.0368
Ad-control+irradiation	Ad-miR-125a-5p	0.9353
Ad-control+irradiation	Ad-miR-125a-5p+irradiation	0.0195
Ad-control+irradiation	Ad-HK2	0.118
Ad-control+irradiation	Ad-HK2+irradiation	0.0028
Ad-control+irradiation	Ad-miR-125a-5p+HK2	0.0305
Ad-control+irradiation	Ad-miR-125a-5p+HK2+irradiation	0.9368
Ad-miR-125a-5p	Ad-miR-125a-5p+irradiation	0.0161
Ad-miR-125a-5p	Ad-HK2	0.113
Ad-miR-125a-5p	Ad-HK2+irradiation	0.0014
Ad-miR-125a-5p	Ad-miR-125a-5p+HK2	0.019
Ad-miR-125a-5p	Ad-miR-125a-5p+HK2+irradiation	0.5949
Ad-miR-125a-5p+irradiation	Ad-HK2	0.0478
Ad-miR-125a-5p+irradiation	Ad-HK2+irradiation	0.0028
Ad-miR-125a-5p+irradiation	Ad-miR-125a-5p+HK2	0.0009
Ad-miR-125a-5p+irradiation	Ad-miR-125a-5p+HK2+irradiation	0.0239
Ad-HK2	Ad-HK2+irradiation	0.2316
Ad-HK2	Ad-miR-125a-5p+HK2	0.2235
Ad-HK2	Ad-miR-125a-5p+HK2+irradiation	0.1215
Ad-HK2+irradiation	Ad-miR-125a-5p+HK2	1
Ad-HK2+irradiation	Ad-miR-125a-5p+HK2+irradiation	0.0094
Ad-miR-125a-5p+HK2	Ad-miR-125a-5p+HK2+irradiation	0.0441
MiR-125a-5p-HK2 WB rH2AX		
Group	Mean	SD
Ad-control	0.66505	0.0069945
Ad-control+irradiation	0.81534	0.019819
Ad-miR-125a-5p	0.77534	0.012628
Ad-miR-125a-5p+irradiation	1.0626	0.021076
Ad-HK2	0.5249	0.028913
Ad-HK2+irradiation	0.68544	0.022689
Ad-miR-125a-5p+HK2	0.64872	0.0059866
Ad-miR-125a-5p+HK2+irradiation	0.83427	0.028686
GroupI	GroupJ	p
Ad-control	Ad-control+irradiation	2.20E-06
Ad-control	Ad-miR-125a-5p	0.0001
Ad-control	Ad-miR-125a-5p+irradiation	1.60E-12

Ad-control	Ad-HK2	5.55E-06
Ad-control	Ad-HK2+irradiation	0.9083
Ad-control	Ad-miR-125a-5p+HK2	0.9692
Ad-control	Ad-miR-125a-5p+HK2+irradiation	4.33E-07
Ad-control+irradiation	Ad-miR-125a-5p	0.2917
Ad-control+irradiation	Ad-miR-125a-5p+irradiation	1.78E-09
Ad-control+irradiation	Ad-HK2	1.44E-10
Ad-control+irradiation	Ad-HK2+irradiation	1.48E-05
Ad-control+irradiation	Ad-miR-125a-5p+HK2	5.37E-07
Ad-control+irradiation	Ad-miR-125a-5p+HK2+irradiation	0.9352
Ad-miR-125a-5p	Ad-miR-125a-5p+irradiation	1.70E-10
Ad-miR-125a-5p	Ad-HK2	1.47E-09
Ad-miR-125a-5p	Ad-HK2+irradiation	0.0011
Ad-miR-125a-5p	Ad-miR-125a-5p+HK2	2.05E-05
Ad-miR-125a-5p	Ad-miR-125a-5p+HK2+irradiation	0.0402
Ad-miR-125a-5p+irradiation	Ad-HK2	2.55E-14
Ad-miR-125a-5p+irradiation	Ad-HK2+irradiation	3.66E-12
Ad-miR-125a-5p+irradiation	Ad-miR-125a-5p+HK2	8.11E-13
Ad-miR-125a-5p+irradiation	Ad-miR-125a-5p+HK2+irradiation	5.88E-09
Ad-HK2	Ad-HK2+irradiation	8.96E-07
Ad-HK2	Ad-miR-125a-5p+HK2	2.71E-05
Ad-HK2	Ad-miR-125a-5p+HK2+irradiation	5.67E-11
Ad-HK2+irradiation	Ad-miR-125a-5p+HK2	0.3849
Ad-HK2+irradiation	Ad-miR-125a-5p+HK2+irradiation	2.50E-06
Ad-miR-125a-5p+HK2	Ad-miR-125a-5p+HK2+irradiation	1.18E-07
